# A randomized control trial evaluating the advice of a physiological-model/digital twin-based decision support system on mechanical ventilation in patients with acute respiratory distress syndrome

**DOI:** 10.3389/fmed.2024.1473629

**Published:** 2024-10-30

**Authors:** Brijesh V. Patel, Sharon Mumby, Nicholas Johnson, Rhodri Handslip, Sunil Patel, Teresa Lee, Martin S. Andersen, Emanuela Falaschetti, Ian M. Adcock, Danny F. McAuley, Masao Takata, Thomas Staudinger, Dan S. Karbing, Matthieu Jabaudon, Peter Schellongowski, Stephen E. Rees, On behalf of the DeVENT Study Group

**Affiliations:** ^1^Division of Anaesthetics, Pain Medicine and Intensive Care, Department of Surgery and Cancer, Faculty of Medicine, Imperial College London, London, United Kingdom; ^2^Department of Critical Care, Royal Brompton Hospital, London, United Kingdom; ^3^Airway Disease, National, Heart and Lung Institute, Imperial College, London, United Kingdom; ^4^Imperial Clinical Trials Unit, Stadium House, London, United Kingdom; ^5^Department of Health Science and Technology, Faculty of Medicine, Aalborg University, Gistrup, Denmark; ^6^Wellcome-Wolfson Institute for Experimental Medicine, Queen’s University, Belfast, United Kingdom; ^7^Department of Medicine I, ICU 13.i2, Medical University of Vienna, Vienna, Austria; ^8^Department of Perioperative Medicine, University Hospital of Clermont-Ferrand, GReD, Université Clermont Auvergne, CNRS, INSERM, Clermont-Ferrand, France

**Keywords:** ARDS, mechanical ventilation, clinical decision support, respiratory mechanics, driving pressure

## Abstract

**Background:**

Acute respiratory distress syndrome (ARDS) is highly heterogeneous, both in its clinical presentation and in the patient’s physiological responses to changes in mechanical ventilator settings, such as PEEP. This study investigates the clinical efficacy of a physiological model-based ventilatory decision support system (DSS) to personalize ventilator therapy in ARDS patients.

**Methods:**

This international, multicenter, randomized, open-label study enrolled patients with ARDS during the COVID-19 pandemic. Patients were randomized to either receive active advice from the DSS (intervention) or standard care without DSS advice (control). The primary outcome was to detect a reduction in average driving pressure between groups. Secondary outcomes included several clinically relevant measures of respiratory physiology, ventilator-free days, time from control mode to support mode, number of changes in ventilator settings per day, percentage of time in control and support mode ventilation, ventilation- and device-related adverse events, and the number of times the advice was followed.

**Results:**

A total of 95 patients were randomized in this study. The DSS showed no significant effect on average driving pressure between groups. However, patients in the intervention arm had a statistically improved oxygenation index when in support mode ventilation (−1.41, 95% CI: −2.76, −0.08; *p* = 0.0370). Additionally, the ventilatory ratio significantly improved in the intervention arm for patients in control mode ventilation (−0.63, 95% CI: −1.08, −0.17, *p* = 0.0068). The application of the DSS led to a significantly increased number of ventilator changes for pressure settings and respiratory frequency.

**Conclusion:**

The use of a physiological model-based decision support system for providing advice on mechanical ventilation in patients with COVID-19 and non-COVID-19 ARDS showed no significant difference in driving pressure as a primary outcome measure. However, the application of approximately 60% of the DSS advice led to improvements in the patient’s physiological state.

**Clinical trial registration:**

clinicaltrials.gov, NCT04115709.

## Introduction

The clinical presentation of acute respiratory distress syndrome (ARDS) is highly heterogeneous, with varying degrees and types of abnormalities in pulmonary gas exchange and mechanics, and treatment response to ventilator adjustments (1, 2). This physiological heterogeneity is evident even within a single etiology, such as coronavirus disease 2019 (COVID-19), emphasizing the need for individualized ventilator management tailored to each patient (3–8). However, ventilator interventions should also maintain consistency by treating physiological phenotypes with similar treatment responses in a homogenous manner (9). Achieving this balance requires a strategy of personalized, yet replicable, ventilatory care grounded in a clear understanding of the patient’s real-time physiology and standardized responses to specific physiological conditions. Currently, evidence suggests that this is not always achieved, with 69% of ventilator settings failing to adhere to evidence-based lung protective ventilation strategies (10).

Decision support systems (DSS) can aid in the process of individualized ventilatory care by providing advice tailored to the patient’s specific physiological state. When these systems are designed to reflect a patient’s individual physiology, they enable care that is both personalized and consistently replicable (9). This is particularly critical in managing complex conditions such as ARDS. The Beacon Care system (Mermaid Care A/S, Nørresundby) is a physiologically-based DSS that provides recommendations for mechanical ventilation adjustments (11).

**Figure 1:**
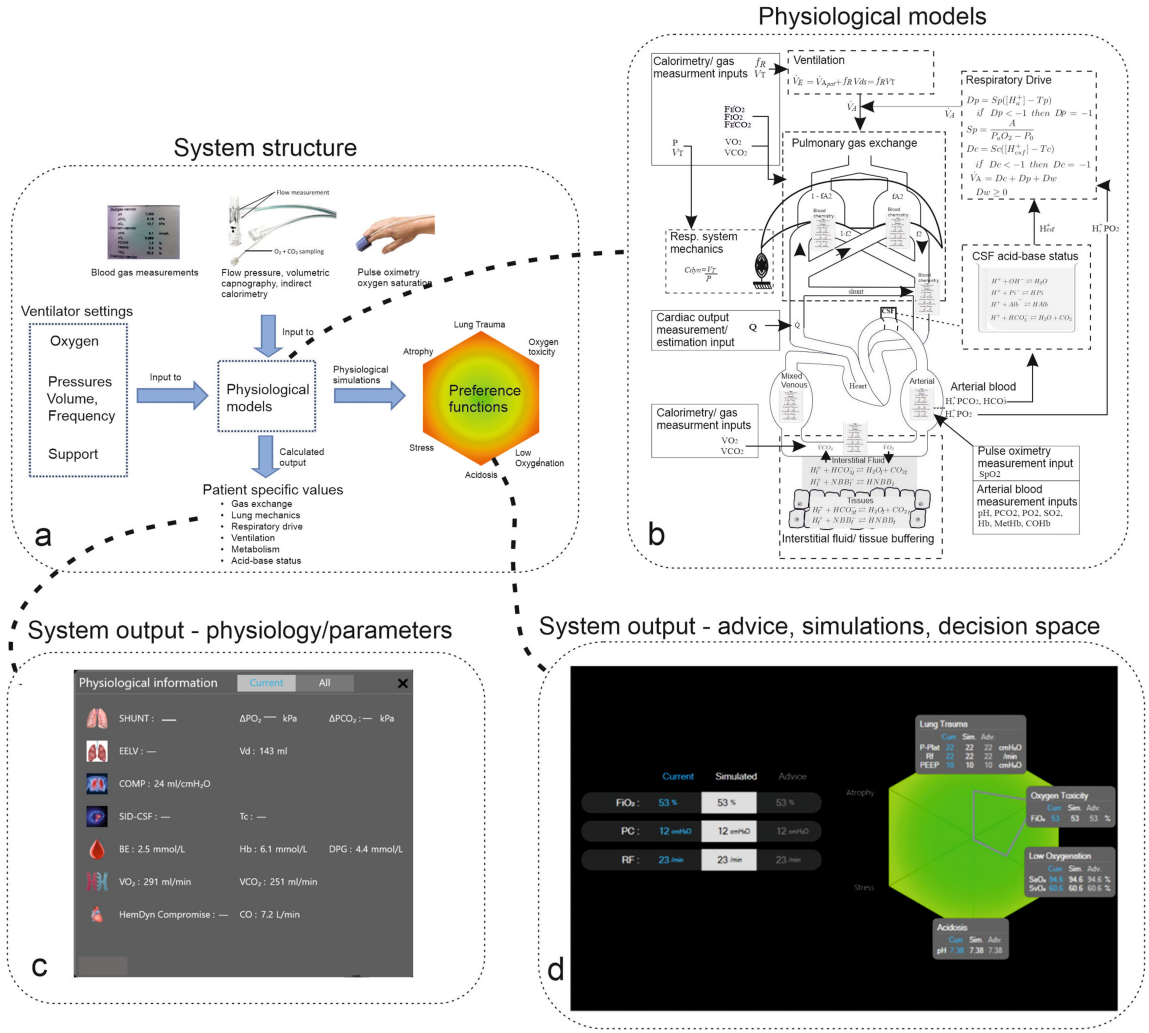
Panel (a) illustrates the structure of the system, including measurement and ventilator inputs to physiological models, which result in calculated patient-specific model parameters and physiological simulations on the color-coded decision space of outcomes. Panel (b) illustrates the complexity of the physiological model, including physiological subsystems (surrounded by dashed boxes) and measurement inputs (surrounded by solid boxes). Panel (c) illustrates the system’s output of patients’ physiological state represented as parameter values for organ systems. Panel (d) illustrates current ventilator settings and systems advice along with the patient’s resulting state, which is illustrated on the hexagon representing the decision space. Gray boxes illustrate current and simulated physiological variables. The green heagon represents a patient with a small risk of adverse effects (color green) with decision-theoretic penalty scores plotted as coordinates on the hexagon represented as a gray outline.

As illustrated in Figure 1 and described in detail in the electronic Supplementary material, this system integrates data from pulmonary mechanics, gas exchange, indirect calorimetry, volumetric capnography, blood acid–base balance, and pulse oximetry. It uses these inputs to calibrate mathematical models of patent physiology, creating a “digital twin” of the patient. This individualized model allows the system to offer recommendations aimed at minimizing the risk of negative outcomes, using utility theory to evaluate trade-offs (12).

The model-tuning process adjusts the generic physiological models based on the patient’s data, making the system adaptable to individual variations. In addition to advice, the system can be used to analyze the patient’s physiological state and changes over time and simulate potential responses to changes in ventilator settings. This advice is presented in two formats: suggested ventilator adjustment and visual representations on a hexagon that illustrate the trade-offs involved, supported by simulations that predict the physiological impact of those adjustments.

This multi-layered presentation allows clinicians to interact with the advice according to their expertise, facilitating smooth integration into clinical practice (13) and providing detailed explanations behind each recommendation. Further details of the system and its advice-generation process are available in the electronic Supplementary material (ESM S.1). The physiological models of the system, as depicted in Figure 1, have been previously validated, including in ARDS patients both with and without COVID-19. In addition, the system has shown its ability to improve physiological outcomes during short periods of mechanical ventilation and reduce pressure support without overstressing respiratory muscles in non-ARDS patients (14, 15).

This multi-center study is the first to investigate the application of the DSS in a population of ARDS patients with varying levels of severity. The primary aim was to determine whether the use of the DSS could positively impact the physiological status of ARDS patients. Additionally, the study sought to evaluate the barriers to the system’s implementation and adoption in clinical settings.

## Materials and methods

### Trial design and oversight

DeVENT was a multi-center, international, randomized, controlled, allocation-concealed, open, pragmatic, superiority clinical trial enrolling patients with ARDS. In the United Kingdom, the study received ethical approval from the London South-East Research Ethics Committee (Ref: 19/LO/1606) on 15th January 2020, with protocol version 4.0 approved on 16th June 2021.

The study protocol was approved by the French Ethics Committee (Comité de Protection des Personnes Sud Mediterranee III; approved on 30th July 2020, under number 2019.12.06 ter_19.11.15.76132) and the French Medicine Agency (Agence Nationale de Sécurité du Médicament; approved on 3th July 2020, under number 2019-A02610-57-A). The study protocol was approved by the ethics committee of the Medical University of Vienna, Austria (EC No. 2056/2019) on 28th April 2020. Monitoring and oversight were provided by the Imperial Clinical Trials Unit and independent trial steering, data monitoring, and ethics committees. The study was conducted in accordance with Good Clinical Practice guidelines, local regulations, and the ethical principles outlined in the Declaration of Helsinki.

### Participants

The study was conducted in three adult intensive care units (ICUs), across the United Kingdom, France, and Austria. Adult patients were eligible for inclusion if they were receiving invasive mechanical ventilation and met the criteria for ARDS as defined by the Berlin definition. These criteria included: the following: a known clinical insult leading to worsening respiratory symptoms, the presence of bilateral infiltrates on a chest radiograph consistent with pulmonary edema not fully explained by cardiac failure, and hypoxemia with a PaO_2_/FiO_2_ ratio of ≤300 mmHg (or ≤ 40 kPa). For patients placed on extracorporeal support, pre-ECMO PaO_2_/FiO_2_ ratios were used (16).

The exclusion criteria included patients younger than 18 years, absence of an arterial catheter, mechanical ventilation lasting longer than 7 days, imminent withdrawal of treatment within 24 h; a do-not-resuscitate (DNR) order, severe chronic respiratory disease requiring home ventilation and/or oxygen therapy (excluding sleep-disordered breathing), requirement of veno-arterial ECMO, and head trauma or other conditions requiring tight regulation of arterial CO_2_ levels. Informed consent was obtained from the patient, a personal consultee, or an independent nominated professional, with retrospective consent obtained from the patient or personal consultee when possible.

### Procedures

The Beacon Care system was attached to all enrolled patients, who were then randomized to either have the system’s advice active (intervention group) or inactive (control group). For patients in the control group, data from the system and driving pressure measurements were not available to the attending physicians. Randomization was stratified by site, ECMO/non-ECMO, and COVID-19/non-COVID-19. The primary objective was to assess whether the use of the DSS affected the driving pressure applied to patients when ventilated in a controlled ventilation mode. Full details of the study protocol have been published previously (17), and the study has been registered on clinicaltrials.gov (NCT04115709). A summary of the methods is provided here, with all other clinical therapies administered according to standard practice.

In the intervention arm, the DSS was attached, and advice was activated. Advice was suspended during periods when the DSS algorithm could not function (e.g., during ECMO or airway pressure release ventilation (APRV), the only ventilation mode not supported by the DSS). Advice continued until extubation, death, or transfer but was not re-initiated on re-intubation. In the control arm, the DSS was attached, and the advice was switched off. A detailed description of the DSS in both arms is provided in the Supplementary material. The research team and nurse superusers were trained by system experts prior to the initiation of the study. Subsequently, the research team conducted regular training and refresher courses, including at-the-elbow training for all new clinical incomers, allowing the system to be operational at all beds.

Data were collected directly from the DSS or entered into an electronic case report form (SMART Trial, Copenhagen, Denmark).

### Outcome measures

The primary outcome measure was average driving pressure over the period of time attached to the DSS, as illustrated in the electronic Supplementary material (ESM S2).

Secondary clinical outcome measures were as follows: (1): daily average calculated delivered pressure over time during periods of spontaneous breathing; (2) daily average calculated mechanical power over time; (3) daily average calculated oxygenation index over time; (4) daily average ventilatory ratio over time; (5) ventilator-free days at 90 days; (6) time from control mode to support mode; (7) a number of changes in ventilator settings per day; (8) a percentage of time in control mode ventilation; (9) a percentage of time in support mode ventilation; (10) total duration of mechanical ventilation; (11) tidal volume over time; (12) PEEP setting over time; (13) ventilation-related complications, e.g., pneumothorax and/or pneumomediastinum; (13) device malfunction event rate; (14) device-related adverse event rate; and (15) number of times the advice from the Beacon system is followed through the duration of the study. All outcomes were reported from the time of randomization. Measurement and/or calculation of outcomes are described in the ESM. The outcomes not reported in this article will be reported separately.

### Statistical analysis

Assuming a standard deviation of 2.5 cmH_2_O and including a 40% dropout, 110 patients would provide 90% power to detect a 2 cmH_2_O reduction in driving pressure. Following the onset of COVID-19, study power was re-estimated, taking into consideration repeated measurements and estimating the intraclass correlation coefficient (ICC) of driving pressure values and the coefficient of variation (CV) for days of data collected (per patient) from available data.

From available data, using an ICC of 0.3 and CV of 0.8, 23 patients per arm allowed for a powered analysis. Driving pressure and other repeated measurements were analyzed using a mixed model approach, including a random clustering effect per patient and fixed-effect covariates for site, ECMO and COVID-19 status, duration of ventilation prior to DSS connection, duration of hospital admission prior to intubation, and number of days in non-ECMO and non-support mode.

Continuous variables were presented as mean (SD) or medians (IQR) if non-normally distributed, and appropriate log transformations were considered where analysis residuals were non-normal. Differences between treatment groups are presented with 95% confidence intervals. Categorical data were presented as numbers and percentages, and any comparisons between the two groups were performed using the Chi-squared or Fisher exact test. An intention-to-treat basis was used for the primary analysis, including all patients randomized into the study, with per-protocol analysis used as a follow-up. All statistical tests were 2-sided, and the significance was set at a *p*-value of <0.05. No imputation was carried out for missing data outside of that undertaken within the mixed-effects model. Similarly, no adjustments have been made for multiple testing. Analyses were performed using SAS version 9.4 software.

A per-protocol subgroup analysis was conducted at randomization based on ECMO/non-ECMO status. An additional post-hoc analysis was added to consider the absence of ECMO treatment during the whole period of DSS application. This was decided to account for the extended ECMO and ventilator durations in COVID-19 patients supported on ECMO, which were uncertain at the time of study design.

## Results

**Figure 2:**
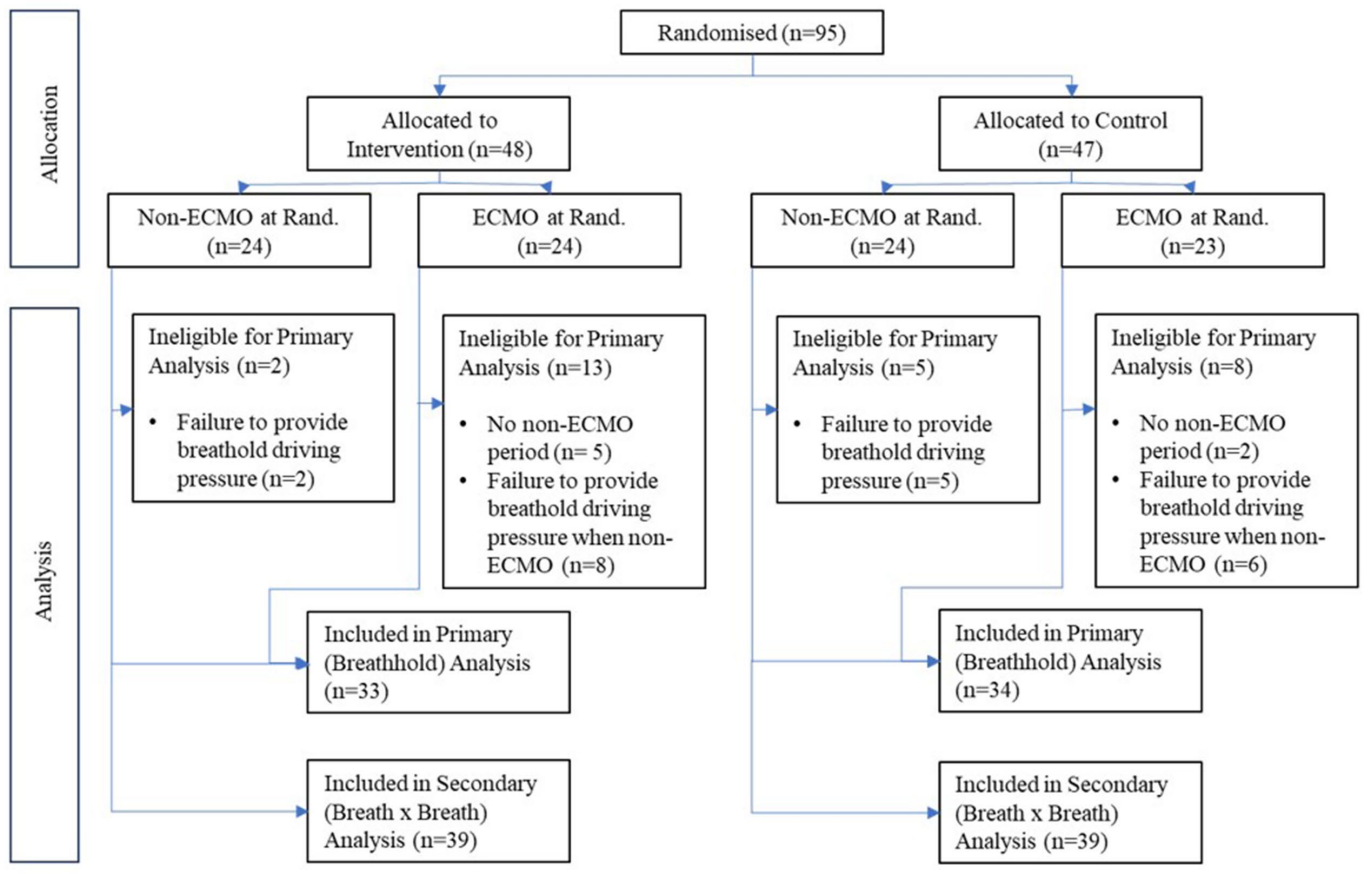
CONSORT diagram illustrating the number of patients randomized in the study and allocated to intervention and control arms and whether treated with extracorporeal membrane oxygenation (ECMO). The number of patients used in each analysis is also illustrated.

Between 19th March 2020 (first patient recruitment) and 4th May 2021, 95 patients fulfilled the inclusion criteria and underwent randomization, with 47 patients allocated to control and 48 patients to the intervention arm (Figure 2). Patients were ventilated in pressure and volume-regulated mandatory modes and pressure support.

Seven patients (two control, five intervention) had no periods off ECMO during their respective follow-up periods, with an additional 21 patients (11 control, 10 intervention) unable to provide a breath-hold driving pressure reading due to either (a) being off ECMO for a minute period (often prior to death/extubation) and/or (b) being exclusively under pressure support during non-ECMO periods. As a result, primary analysis was possible in 67 patients (33 intervention, 34 control). Secondary outcomes based on continuous breath-by-breath measurements could be calculated in 78 patients.

### Patient baseline demographics

Demographic data by study arm are illustrated in Table 1, and treatment groups were well matched for clinical parameters at baseline. Each arm had an almost identical distribution of ECMO and non-ECMO patients.

**Table 1:** Patient demographics at randomization.

Variable		Control (*N* = 47)	Intervention (*N* = 48)	All (*N* = 95)
Age at consent (y)	Mean (SD)	52 (14)	54 (16)	53 (15)
Gender – Male (%)	*n* (%)	30 (64)	32 (67)	62 (65)
Height (cm)	Mean (SD)	172 (9)	171 (9)	172 (9)
Weight (kg)	Mean (SD)	96 (30)	98 (33)	97 (32)
Body mass index (kg/m_2_)	Mean (SD)	33 (10)	33 (11)	33 (10)
Predicted weight (kg)	Mean (SD)	66 (10)	65 (10)	66 (10)
Smoking history (%)	Current smoker	4 (9)	0	4 (4)
	Current vaper	0	1 (2)	1 (1)
	Ex-Smoker	7 (15)	8 (17)	15 (16)
	Never Smoker	14 (30)	20 (42)	34 (36)
	Unknown	22 (47)	19 (40)	41 (43)
Comorbidities – chronic pulmonary disease (%)	*n* (%)	13 (28)	13 (27)	26 (27)
	Unknown	1 (2)	0	1 (1)
Comorbidities – cardiovascular disease (%)	*n* (%)	23 (49)	18 (38)	41 (43)
	Unknown	1 (2)	0	1 (1)
Comorbidities – metabolic and endocrine	*n* (%)	26 (55)	20 (42)	46 (48)
	Unknown	2 (4)	0	2 (2)
Comorbidities – chronic immuno-suppression	*n* (%)	3 (6)	3 (6)	6 (6)
	Unknown	0	1 (2)	1 (1)
Comorbidities – chronic neurological disease	*n* (%)	2 (4)	1 (2)	3 (3)
	Unknown	7 (15)	2 (4)	9 (10)
Days since Ards Onset	Median [IQR]	2 [1–5]	1 [1–3]	2 [1–4]

### Outcomes

**Figure 3:**
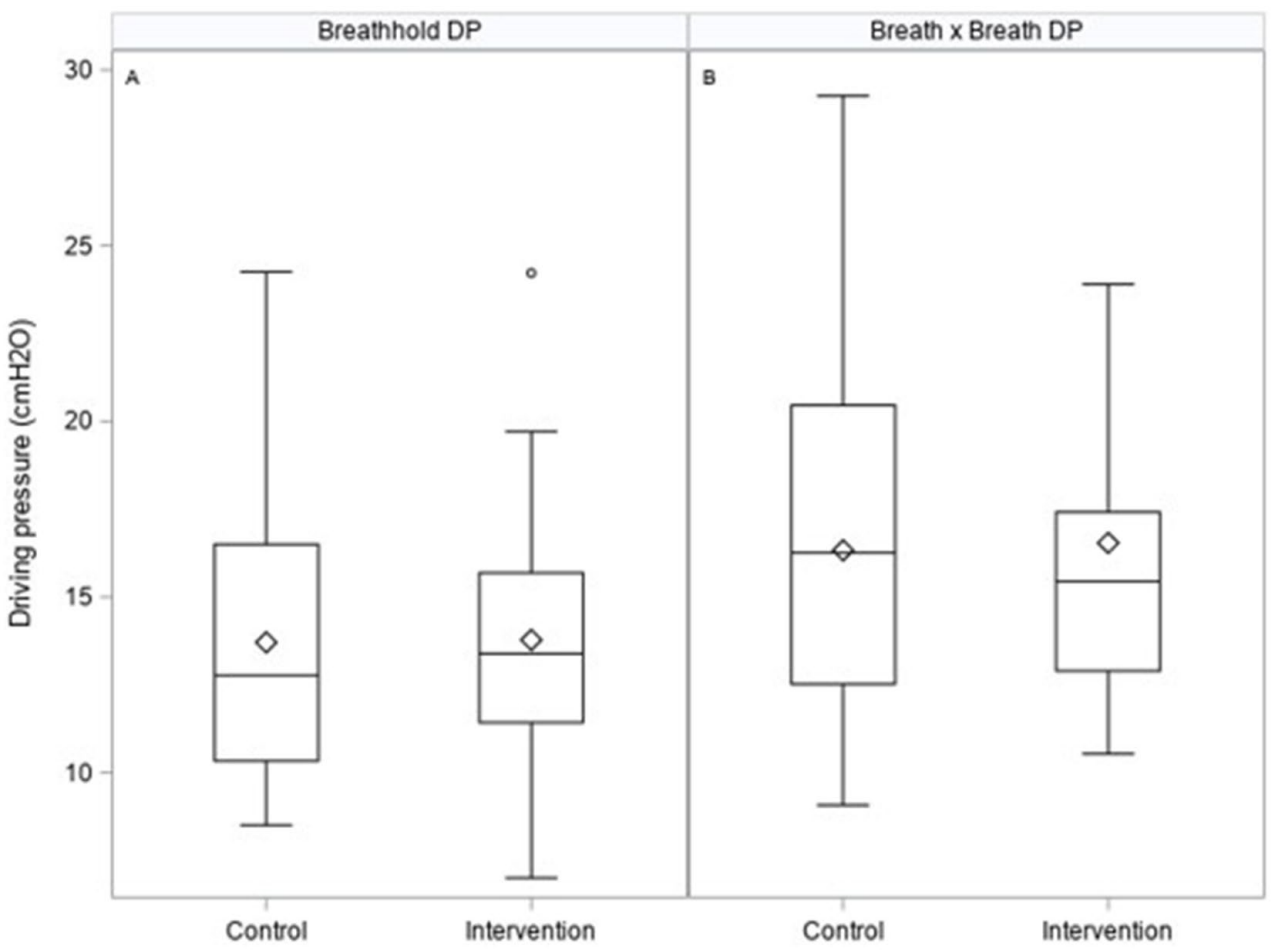
Box plots illustrating primary outcome measures of driving pressures on the control and intervention arm measured from breath holds **(A)** or breath by breath **(B)** as calculated in the ESM.

There was no statistically significant difference in the primary outcome variable, with values of driving pressure (Table 2; Figure 3) measured from either breath hold (−0.34 cmH_2_O with intervention, 95% CI: −2.22, 1.54 cmH_2_O; *p* = 0.72) or continuous measures (−0.23 cmH_2_O, 95% CI: −2.1, 1.67 cmH_2_O; *p* = 0.81) being not statistically different between groups. However, the intervention arm appeared to have some tendency toward tighter interquartile ranges (Figure 3).

**Table 2:** Primary and secondary outcomes, all patients.

	Control		Intervention			
	*n*	mean (SD) median [IQR]	n	mean (SD) median [IQR]	Effect [95% CI]	*p*
Primary outcome measure						
Breath hold driving pressure (cmH_2_O)	34	13.71 (4.20)	33	13.78 (3.62)	−0.34 [−2.22, 1.54]	0.72
Secondary outcome measures						
Breath by breath driving pressure (cmH_2_O)	39	16.33 (4.66)	39	16.54 (4.89)	−0.23 [−2.13, 1.67]	0.81
Breath by breath mechanical power (J/min)	41	24.64 (8.45)	39	25.80 (7.54)	−2.23 [−3.06, 2.60]	0.87
Oxygenation index – mandatory modes	26	11.10 (5.70)	30	11.58 (5.61)	0.33 [−2.34, 3.00]	0.81
Oxygenation index – support modes	24	7.69 (3.49)	25	6.60 (2.39)	−1.41 [−2.74, −0.08]	0.0370
Ventilatory ratio – mandatory modes	26	2.66 (0.82)	31	2.42 (0.59)	−0.31 [−0.67, 0.05]	0.09
Ventilatory ratio – support modes	25	2.55 (0.65)	25	2.56 (0.72)	−0.13 [−0.47, 0.22]	0.48
Average ventilator free days at 90 days^Ʈ^	47	49 [0–73]	48	37.5 [0–69]	0.0 [−20.0, 0.0]	0.27
Time from control to support ~	34	2.35 (3.13)	32	2.42 (2.98)	−0.17 [−1.58, 1.25]	0.81
Average daily settings change (PEEP) ^Ʈ^	44	1.11 [0.54–1.84]	42	1.27 [0.47–2.20]	0.14 [−0.29, 0.58]	0.52
Average daily settings change (FiO_2_) ^Ʈ^	44	7.50 [4.42–10.62]	42	8.27 [4.32–11.7]	1.01 [−1.07, 3.32]	0.29
Average daily settings change (PS) ^Ʈ^	43	2.65 [0.44–5.66]	41	1.23 [0.24–2.70]	−0.84 [−2.10, 0.00]	0.06
Average daily settings change (PC) ^Ʈ^	44	0.66 [0–2.36]	42	2.25 [0–4.86]	0.96 [0.00, 2.15]	0.0246
Average daily settings change (RF) ^Ʈ^	44	0.94 [0–2.02]	42	2.31 [0.95–4.96]	1.30 [0.61, 2.30]	0.0004
% in control mode~	44	55.9 (36.4)	42	50.3 (32.9)	−6.27 [−20.9, 8.33]	0.40
% in support mode~	44	31.3 (32.0)	42	42.1 (31.1)	11.6 [−1.81, 25.0]	0.09
Duration of mechanical ventilation.~	44	10.52 (9.18)	42	13.80 (13.40)	3.04 [−1.35, 7.43]	0.18
Tidal volume measured control	28	531 (113)	33	520 (132)	−10.6 [−74.4, 53.1]	0.74
Tidal volume measured support	27	644 (142)	29	648 (189)	−42.82 [−125.0, 39.4]	0.31
PEEP setting	37	9.85 (2.38)	37	9.88 (2.30)	0.37 [−0.68, 1.42]	0.49
Subjects with device related adverse events ^Ǫ^	47	0 (0%)	48	1 (2%)		1.00
Device-related adverse event rate (per day) ^Ʈ^	47	0 [0–0]	48	0 [0–0]	0.0 [0.0, 0.0]	0.32
Subjects with re-intubation events ^Ǫ^	47	2 (2%)	48	2 (2%)		1.00
percentage time disconnected prior to reintubation^Ʈ^	47	0 [0–0]	48	0 [0–0]	0.0 [0.0, 0.0]	0.98

The table shows the mean (SD) or median [IQR] of primary and secondary outcome variables by treatment group alongside the treatment effect derived from the mixed-effects model. The model incorporates fixed effects for COVID-19 and Site and also adjusted for continuous variables (1) Duration of ventilation prior to connection, (2) duration of hospitalization prior to intubation, and (3) the number of days non-ECMO and non-support and has a random effect accounting for clustering per subject. ~ No clustering effect was included for these variables, as data was provided as an overall average per subject. *p < 0.05, **p < 0.01, ***p < 0.001. Ʈ Median [Q1-Q3] presented. Effect represents the difference in median via Hodges-Lehmann estimate and distribution-free confidence interval. *p*- value from the Wilcoxon Test. Ǫ Frequencies presented as n (%). *p*- values to assess differences in event frequencies derived from Fisher’s exact test. PEEP, Positive end-expiratory pressure; FiO_2,_ fraction inspired oxygen; PS, pressure support; PC, pressure control; RF, respiratory frequency.

Patients in the intervention arm had statistically improved oxygenation index when in support mode ventilation (−1.41, 95% CI: −2.76, −0.08; *p* = 0.0370). For the subsequent post-hoc sub-group analysis of non-ECMO patients (Table 3), the oxygenation index was improved in the intervention arm for patients in support mode (−2.60, 95% CI:-4.13, −1.08; *p* = 0.0010), with controlled mandatory ventilation showing a numerical improvement although this did not reach statistical significance (−2.66, 95% CI -5.38, 0.06; *p* = 0.06). The ventilatory ratio was also significantly improved in the intervention arm for non-ECMO patients in control mode ventilation (−0.63, 95% CI: −1.08, −0.17, *p* = 0.0068), although this effect was reduced when extended to the full study population (−0.31, 95% CI -0.67, 0.05; *p* = 0.09). There is a tendency for patients in the intervention arm to spend a greater proportion of ventilator time in pressure support mode in comparison to mandatory modes (11.6, 95% CI, −1.8, 25.0, *p* = 0.09).

**Table 3:** Primary and secondary outcomes, non-ECMO patients only.

	Control		Intervention			
	*n*	mean (SD)	*n*	mean (SD)	Effect [95% CI]	*p*
Pressure, oxygenation, and ventilation						
Breath hold driving pressure (cmH_2_O)	19	14.79 (4.82)	17	13.64 (3.57)	−1.44 [−3.98, 1.11]	0.27
Secondary outcome measures						
Breath by breath driving pressure (cmH_2_O)	22	17.61 (4.97)	17	18.04 (5.85)	−0.4 [−3.55, 2.27]	0.67
Breath by breath mechanical power (J/min)	23	26.91 (8.88)	17	27.88 (8.71)	−1.22 [−5.08, 2.63]	0.53
Oxygenation index – mandatory modes	17	13.14 (5.89)	14	12.05 (5.81)	−2.66 [−5.38, 0.06]	0.06
Oxygenation index – support modes	12	9.11 (6.08)	11	6.08 (2.13)	−2.60 [−4.13, −1.08]	0.0010
Ventilatory ratio – mandatory modes	17	2.72 (0.85)	14	2.24 (0.52)	−0.63 [−1.08, −0.17]	0.0068
Ventilatory ratio – support modes	13	2.64 (0.1)	11	2.27 (0.50)	−0.13 [−0.47, 0.22]	0.48
Average ventilator free days at 90 days^Ʈ^	23	19 [9–29]	19	16 [9–27]	0.0 [−13.0, 12.0]	0.89
Time from control to support ~	17	3.28 (3.94)	15	3.88 (3.45)	0.32 [−2.45, 3.09]	0.82
Average daily settings change (PEEP) ^Ʈ^	23	1.41 [0.83–3.20]	19	1.37 [1.05–2.20]	−0.16 [−1.20, 0.55]	0.69
Average daily settings change (FiO_2_) ^Ʈ^	21	8.51 [3.16–11.65]	16	9.30 [3.02–11.63]	0.12 [−3.83, 3.21]	0.92
Average daily settings change (PS) ^Ʈ^	23	2.10 [0.20–5.66]	18	2.06 [1.00–3.06]	−0.22 [−2.10, 1.00]	0.64
Average daily settings change (PC) ^Ʈ^	23	1.17 [0–1.96]	19	2.31 [0–5.56]	0.26 [−0.48, 2.41]	0.37
Average daily settings change (RF) ^Ʈ^	20	1.40 [0.70–2.27]	19	2.33 [0.96–4.24]	0.92 [0.00, 2.22]	0.06
% in Control Mode~	23	63.2 (32.8)	19	49.3 (30.2)	−13.39 [−32.82, 6.05]	0.17
% in Support Mode~	23	29.0 (29.6)	19	41.8 (30.4)	12.75 [−5.70, 31.2]	0.17
Duration of mechanical ventilation~	23	13.07 (9.24)	19	15.13 (9.45)	0.99 [−4.71, 6.70]	0.73
Tidal volume measured control	17	517 (130)	14	499 (113)	27.1 [−50.5, 104.8]	0.49
Tidal volume measured support	13	658 (168)	11	621 (140)	−7.90 [−121.2, 105.4]	0.89
PEEP setting	21	10.31 (2.61)	16	10.20 (2.93)	0.35 [−1.07, 1.76]	0.63
Subjects with device related adverse events^Ǫ^	23	0 (0%)	19	1 (5%)		0.45
Device-related adverse event rate (per day)^Ʈ^	23	0 [0–0]	19	0 [0–0]	0.0 [0.0, 0.0]	0.27
Subjects with Re-intubation events ^Ǫ^	23	2 (9%)	19	2 (11%)		1.00
percentage time disconnected prior to reintubation^Ʈ^	23	0 [0–0]	19	0 [0–0]	0.0 [0.0, 0.0]	0.84

Post-hoc analysis exclusively looked at patients who were never placed on ECMO. The table displays the mean (SD) or median [IQR] of primary and secondary outcome variables by treatment group alongside the treatment effect derived from the mixed-effects model. The model incorporates fixed effects for COVID-19 and Site and also adjusted for continuous variables (1) Duration of Ventilation prior to connection, (2) duration of hospitalization prior to intubation, and (3) the number of days non-ECMO and non-support and has a random effect accounting for clustering per subject. ~ No clustering effect was included for these variables, as data were provided as an overall average per subject. *p < 0.05, **p < 0.01, ***p < 0.001. Ʈ Median [Q1-Q3] presented. Effect represents the difference in median via Hodges-Lehmann estimate and distribution-free confidence interval. *p*- value from Wilcoxon Test. Ǫ Frequencies presented as n (%). *p*- values to assess differences in event frequencies derived from the Fisher’s exact test. PEEP, Positive end-expiratory pressure; FiO_2_, fraction inspired oxygen; PS, pressure support; PC, pressure control; RF, respiratory frequency.

**Figure 4:**
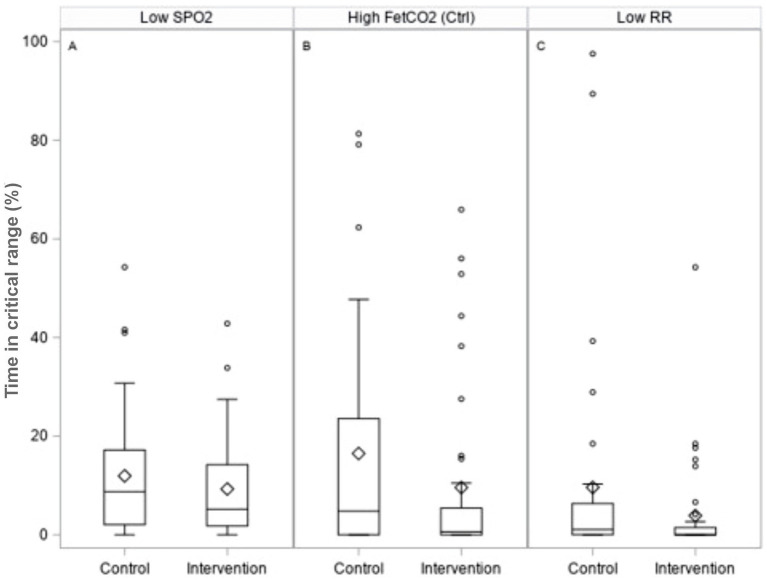
Box plots illustrating safety measures for the percentage time spent: A - below SpO_2_ values of 88%; B - above end-tidal CO_2_ values of 7 kPa in control ventilation; and C - below the respiratory rate of 12 breaths per minute in pressure support ventilation.

Safety data are presented in Table 4 and Figure 4. No significant differences were observed in median time-to-death (control vs. intervention: 19 (15–59) vs. 19.5 (10–36) days; *p* = 0.64).

**Table 4:** Safety analysis showing a 90-day mortality rate and key ventilation parameters.

	Control			Intervention			Comparison	
		Deaths	Days to event		Deaths	Days to event		
	*n*	*n* (%)	Med. [Q1-Q3]	*n*	*n* (%)	Med. [Q1-Q3]	Δ [95% CI]	*p*- value
Death – all patients	47	12 (26)	19.0 [15–59]	48	18 (38)	19.5 [10–36]	−1.5 [−18.0, 14.0]	0.64
Death – non-ECMO patients	23	9 (39)	19.0 [18–22]	19	7 (37)	16.0 [6–21]	−6.0 [−44.0, 5.0]	0.20
	*n*	Mean	Median	*n*	Mean	Median	Δ [95% CI]	*p*- value
% time below SpO_2_ 88%	44	12.0	8.7	42	9.3	5.2	**−0.98 [−5.07, 1.51]**	0.44
% time FetCO_2_ > 7 kPa, mandatory	32	16.5	4.8	38	9.6	0.6	**−0.05 [−7.96, 0.00]**	0.19
% time FetCO_2_ > 7 kPa, support	32	16.7	0.1	34	7.4	0.0	**0.00 [−1.06, 0.00]**	**0.06**
% time RF < 12, support	34	9.6	1.1	35	3.9	0.0	−0.20 [−1.58, 0.00] *	**0.0167**
% time RSBI >100, support	32	1.0	0.0	34	0.8	0.0	**0.00 [0.00, 0.00]**	0.78

Δ [95% CI] represents the difference in medians and a distribution-free 95% CI via Hodges-Lehmann estimate and distribution-free confidence interval. *p*- value from Wilcoxon Test. *p < 0.05; **p < 0.01; ***p < 0.001. SpO_2_, pulse oximetry oxygen saturation; FetCO_2_, end-tidal carbon dioxide fraction; RF, respiratory frequency; RSBI, rapid shallow breathing index.

We observed no significant difference in mortality between the intervention arm (38% vs. 26%) and the subset of patients not receiving ECMO (37% vs. 39%). There were no differences between ventilation-related complications, device malfunction rates, or device-related adverse events. No significant difference was seen in the time spent or incidence of hypoxemia or hypercapnia in patients ventilated on control mode between groups. In support mode, in terms of per-patient percentage of their respective DSS application, significantly less time was spent at very low respiratory rates (< 12 breaths per minute) in the intervention arm (difference in medians −0.2, 95% CI -1.6, 0.0; *p* = 0.0167), and at high CO_2_ levels (>7 kPa), although this was not statistically significant (difference in medians −0.0, 95% CI -1.1, 0.0; *p* = 0.06; Figure 4; Table 4).

The application of the DSS resulted in a significantly increased number of ventilator changes for pressure settings during control mode ventilation (control vs. intervention: 0.65 vs. 2.25 changes per day; *p* < 0.05) and respiratory frequency (control vs. intervention: 0.94 vs. 2.31 changes per day; *p* < 0.001). Table 5 illustrates the uptake of advice over a 2-h window following the advice presentation, where advice was to change ventilator settings to those different from current values. Indeed, only 44% of advice was given to change pressure support, and 66% for FIO_2_ was actioned. The advice was often not followed, meaning that no ventilator changes were made during the two-hour window following the advice presentation. Of note, changes in ventilator settings in the opposite direction to the advice presented within the two-hour window were minimal.

**Table 5:** Application of system’s advice – intervention arm only.

		Advice Followed (%)	Advice Ignored (%)	Changes made which were opposite to the advice suggested (%)
	n	Median [Q1-Q3]	Median [Q1-Q3]	Median [Q1-Q3]
FiO_2_, PEEP	34	65.7 [40.0–72.7]	25.0 [12.5–40.0]	12.1 [0.0–0.0]
PC, RF (mandatory)	19	60.0 [0.0–71.4]	33.3 [13.3–83.3]	0.0 [0.0–14.3]
VT, RF (mandatory)	7	61.5 [14.3–83.3]	50.0 [9.5–66.5]	16.7 [4.8–50.0]
PS	21	43.8 [0–60.0]	39.6 [0.0–54.5]	0.0 [0.0–10.0]

The table presents medians and quartiles of how the advice was implemented following Beacon recommendations. Advice recommended by Beacon was either followed, ignored (with no change made to ventilator settings), or different from what the clinician advised. FiO_2_, fraction inspired oxygen; PEEP, Positive end-expiratory pressure; PC, pressure control; RF, respiratory frequency; PS, pressure support.

No other significant differences were seen in secondary endpoints assessing physiological measurements or ventilation duration.

## Discussion

This study was designed to evaluate this decision support system in patients with ARDS receiving mechanical ventilation prior to COVID-19 onset and repurposed for implementation during the pandemic under extremely challenging circumstances, with the inclusion of patients with ARDS from both COVID-19 and non-COVID-19 etiologies. This DSS is a novel, open-loop system providing advice from physiological mathematical models individualized by automatically tuning models to the patient’s data at the bedside. These systems aid rather than replace clinical expertise and provide personalized, adapted therapy as the models learn from changes in patient state and exemplars of recently described systems for the future (18).

No significant differences were seen in driving pressure, the study’s primary outcome; however, patients in the intervention arm tended to have tighter regulation of driving pressure than those in standard care. This may be important as it is perhaps more crucial to reduce the incidence of high levels of driving pressure than to modify the median value delivered, as values below 15cmH_2_O may reflect little increased risk for the patient (19).

Application of DSS advice showed optimization of physiological state under certain conditions. Significant improvement in oxygenation index was seen without increased incidence of hypoxemia, consistent with appropriate use of FiO_2_. A significantly improved ventilatory ratio was seen for non-ECMO patients under controlled ventilation modes.

We observed a significantly greater number of changes in pressure control (2.25 vs. 0.66) and respiratory frequency settings (2.31 vs. 0.94) in the intervention arm, but this was not significant in non-ECMO patients (2.31 vs. 1.17 and 2.33 vs. 1.30, respectively), and no significant changes in the number of pressure support setting changes. Support mode ventilation was delivered at significantly reduced time spent with a respiratory rate of less than 12 breaths/min, values below which have been shown previously to be associated with ventilatory over assistance (20). Furthermore, there was a trend toward a reduction in the percentage time spent at end-tidal carbon dioxide levels greater than 7 kPa (*p* = 0.06), alongside non-significant tendencies for pressure support reduction of about 1-2cmH_2_O. No significant detrimental effects were observed by the application of the advice.

Despite the challenges of implementing a complex, “open loop” DSS intervention during pandemic conditions, the data indicate that advice was applied approximately 60% of the time, with the exception of advice on pressure settings, for which advice was applied a median of 44%. However, despite a significant proportion of advice being either ignored or not seen, optimization of gas exchange still occurred. This may reflect the system requesting small changes in ventilatory parameters, which may be at odds with purely clinician-made changes. This lack of adoption may be enhanced during the pandemic surges and contrasts with other studies evaluating the application of the system in short, controlled periods (14, 15). Importantly, for advice on pressure settings in either mandatory or support modes, clinicians tended not to make changes different from the advice (as represented by a median change count of zero within both mode settings). For FiO_2_, clinicians made settings in the opposite direction a median of 12% of the time. The lack of implementation of a higher percentage of advice places limitations on the interpretation of these results, illustrating that improvements may be made when the advice is used as an augmentation of current care rather than a replacement. If such a system were to be used in a closed-loop context, analysis of the reasons for differences in opinions would be necessary. Nonetheless, this system also enables an understanding of the frequency of ventilator changes that are made and the implementation space for such a device.

Other decision support systems and closed-loop control systems for mechanical ventilation have been evaluated in prospective studies, but few in relation to the management of ARDS and none with a detailed physiological model of the individual patient. Notably, East et al. showed physiological efficacy through significant improvement in morbidity scores in 200 ARDS patients randomized to decision support advice based on empirical rules rather than physiological models in a non-commercial system (21). The most widely applied and evaluated routine commercial tools, SmartCare (Dräger Medical) and Intelli-Vent Adaptive Support Ventilation (INTELLiVENT-ASV) or its predecessor ASV (Hamilton Medical), apply closed-loop automation rather than open-loop advice. SmartCare provides control during support mode ventilation and has been shown in some studies to significantly reduce weaning duration in patients after evaluation of weaning readiness (22, 23). ASV and INTELLiVENT-ASV control the patient through all phases of ventilation, and studies have shown significant reductions in weaning time (24, 25) and total ventilator time (26, 27). However, these have been in fast-track cardiac patients or patients with COPD, but patients with ARDS have been excluded. A single retrospective study with Intelli-Vent ASV has shown a significant reduction in driving pressure in 51 COVID-19-ARDS patients 2 h after conversion to INTELLiVENT-ASV, suggesting potential for improvement in care without evidence provided by prospective evaluation (28). Hence, such novel technologies using personalized approaches may lead to improvements in weaning (29).

Titration of ventilator settings in the acute phase based on pressure-volume curves and applying optimal PEEP and driving pressure settings are associated with better outcomes in ARDS (30, 31). However, it remains uncertain whether this benefit is due to optimized PEEP or the use of low tidal volume ventilation (32). Indeed, within the current era of ARDS, lung protective ventilation strategies remain a standard of care in the specialist center involved in this study and may explain why no effect on driving pressure was apparent. Furthermore, the differences in pathophysiology between COVID-19-ARDS and non-COVID-19 ARDS may have played a role in how the system could influence reductions in driving pressure and may explain why differences were observed in composite measures such as oxygenation index and ventilatory ratio (33–36).

There are several limitations to this study. The primary challenge was conducting the research during the COVID-19 pandemic, which created a less-than-ideal environment for the evaluation and implementation of a new device. This likely contributed to the high percentage of advice being ignored by clinicians. In addition, two of the study sites were ECMO centers, and many of the enrolled patients required ECMO support, which introduced greater heterogeneity in the patient cohort. The device in this study measures oxygen consumption and carbon dioxide production at the mouth using indirect calorimetry and incorporates these data into its advice calculations. However, it does not account for the gas exchange occurring through ECMO, making its use contraindicated during ECMO periods.

Adapting the device to account for ECMO gas exchange would, in the future, enable the calculation of advice during ECMO periods. The evaluation of differences in driving pressure in patients who underwent a few days of non-ECMO ventilation, followed by a prolonged ECMO period, and then another period of non-ECMO ventilation, may have resulted in comparisons biased by the duration of ECMO. Notably, our sensitivity analysis showed that improvements in physiological status were more pronounced in the non-ECMO group.

## Conclusion

This study is the first evaluation of a physiological model-based DDS for guiding mechanical ventilation in patients with both COVID-19 and non-COVID-19 ARDS. The results showed no significant difference in driving pressure as the primary outcome. However, the application of approximately 60% of the system’s advice led to improvements in the physiological state. The study also demonstrated greater homogeneity in ventilatory management, and the system proved safe to implement in patients with ARDS, even during pandemic conditions. Further studies assesing the implementation of such a DSS would help determine its clinical significance and/or health economic benefits.

## Group member of DeVENT Study Group

See Supplementary material.

## Data Availability

The datasets presented in this article are not readily available because the datasets generated during the current study are not publicly available due to privacy issues but may be available from the corresponding author on reasonable request conditional upon local law relating to confidentiality and anonymization. Requests to access the datasets should be directed to sr@hst.aau.dk.
